# Effect of Japanese Kampo Medicine Therapy for Menopausal Symptoms after Treatment of Gynecological Malignancy

**DOI:** 10.1155/2018/9475919

**Published:** 2018-04-02

**Authors:** Akihiko Yoshimura, Kenjiro Sawada, Tomoyuki Sasano, Hiromasa Kuroda, Katsumi Kozasa, Erika Nakatsuka, Koji Nakamura, Kae Hashimoto, Seiji Mabuchi, Tadashi Kimura

**Affiliations:** Department of Obstetrics and Gynecology, Osaka University Graduate School of Medicine, 2-2 Yamadaoka, Suita, Osaka 565-0871, Japan

## Abstract

Loss of ovarian function by the treatment for gynecological malignancy results in a drastic decrease of estrogen causing physical and mental symptoms. The purpose of this study is to evaluate the effect of Japanese Kampo Kamikihito (KKT) and Kamishoyosan (KSS) on menopausal symptoms in gynecological cancer patients. Patients who had menopausal symptoms after gynecologic malignancy treatment were enrolled and randomly divided into a KKT or a KSS group. Kupperman Menopausal Index (KI) questionnaires were obtained before tumor treatment, at baseline, and at 4 and 8 weeks. Changes in KI scores and severity of each symptom were evaluated. A total of 33 patients were enrolled: 18 in the KKT group and 15 in the KSS group. The KI scores significantly decreased at 4 and 8 weeks compared with baseline in both groups. Although no significant difference was found in change in KI scores between the KKT and KSS groups, efficacy showed some differences. Both KKT and KSS were effective for insomnia, vertigo, and palpitation. KSS was also effective for vasomotor symptoms and arthralgia/myalgia. In conclusion, both KKT and KSS were effective for menopausal symptoms in patients after gynecological tumor treatment. Tailor-made Kampo therapy may contribute to improve patients' physical and mental symptoms.

## 1. Introduction

The number of patients with gynecological malignant tumors is increasing every year. In the treatment for gynecological malignancy, various treatments such as oophorectomy, cytotoxic chemotherapy, and pelvic radiotherapy are used, and as a result of these treatments, the loss of ovarian function is inevitable in most cases [1]. Loss of ovarian function results in a decrease of estrogen causing physical and mental symptoms such as hot flashes, nocturnal sweating, anxiety, irritability, and impairment of QOL. These symptoms are similar to menopausal disorders, and efficacy can be expected with hormone replacement therapy (HRT). However, the safety of HRT has not been established in patients receiving treatment for estrogen-dependent tumors such as in endometrial cancer [1–3]. Besides, patients often decline HRT because of their concerns about the risk of thrombosis or breast cancer in Japan. Thus, there has been no “gold standard” for treating these mental and physical symptoms that arise from treatment for a malignant tumor.

In Japan, Kampo medicine (Kampo) therapy is often used for patients with general malaise. Patients with menopausal symptoms arising from the loss of ovarian function are often prescribed tailor-made Kampo [4, 5]. However, the comprehensive therapeutic effect of Kampo for ovarian function loss due to anticancer treatment has not been elucidated. Previously, we reported on the effectiveness of Kamikihito (KKT) for menopausal symptoms associated with gonadotropin-releasing hormone (GnRH) agonist treatment [6]. KKT has been adapted for patients with a weak constitution such as anemia, insomnia, anxiety, and neurosis, and efficacy has been reported for the general malaise of menopausal disorders [7, 8]. Experimental studies of KKT have shown pharmacological effect on autonomic imbalance [9–12] and insomnia [13]. Thus, efficacy can be expected for the various symptoms following the loss of ovarian function. Kamishoyosan (KSS) is commonly used by Japanese gynecologists [14], and its efficacy for general malaise associated with menopausal disorder has been reported [15]. However, there has been no consensus regarding which Kampo to prescribe. Therefore, we conducted this study to evaluate the effect of KKT and KSS on menopausal symptoms in patients who had received treatment for gynecological malignancy.

## 2. Materials and Methods

### 2.1. Patients

Patients who had menopausal symptoms while undergoing treatment for gynecological malignant tumors at the Osaka University Hospital from November 2012 to December 2015 were enrolled. They scored the Kupperman Menopausal Index (KI): Abe modification (Table 1) [16], and weighted index score was calculated. With this scoring system, minimum score is 0 and maximum weighted index is 51. The interpretation of severity is as follows: none: 0; minimal: 1 to 14; mild: 15 to 20; moderate: 21 to 35; and severe: 36 to 51. Thus, the inclusion criterion was a total score of 21 or over on KI scores after the treatment of gynecologic malignancy, as their symptoms are defined as moderate to severe. Exclusion criteria were as follows: (1) patients receiving Kampo, herbal preparations, or HRT and (2) patients with a history of or who were suspected of having aldosteronism, myopathy, or hypokalemia. Written informed consent was obtained from all patients. This study complied with the Declaration of Helsinki and was implemented with the approval of the Osaka University Ethics Committee.

### 2.2. Methods

A randomized parallel group study was conducted. Patients were randomly divided into the KKT group or the KSS group using a simple envelope randomization method. After patients' consent was obtained, numbered envelopes which included group name were opened at the data office. Thereafter, each treatment group inside the envelope was addressed to researchers. A KI questionnaire survey was conducted by researchers at outpatient clinic on subjective symptoms before tumor treatment, at baseline (before Kampo treatment), at 4 weeks, and at 8 weeks. Kampo treatment was started within four weeks after the treatment against gynecological malignancies had been finished. Chronological changes in KI scores were evaluated in both groups. As a primary endpoint, changes in KI scores (ΔKI) between baseline and 8 weeks were compared between the groups. As a secondary endpoint, changes in the severity scores of each subitem listed in the KI between baseline and 8 weeks were also compared between groups. For the safety evaluation, adverse events (AEs) were collected. Patient background including age, height, weight, medical history, and complications was collected. The study drugs were Kracie Kamikihito Extract Fine Granules, 7.5 g (KKT) (Kracie Pharma, Ltd., Tokyo, Japan) and Kracie Kamishoyosan Extract Fine Granules, 6.0 g (KSS) (Kracie Pharma, Ltd.). Study drugs were administered twice a day before or with a meal for 8 weeks. In principle, medications other than study drugs were continued without dose modification during the study.

### 2.3. Statistics

Friedman tests and Shaffer's multiple comparisons were performed to evaluate changes in KI scores. The Mann–Whitney *U* test was used to compare ΔKI between the KKT and KSS groups. Wilcoxon signed-rank tests were performed to evaluate the change in the scores of the subitems of the KI. Alpha was set at 0.05, two-sided. Statcel 3 software (OMS Publishing Inc., Saitama, Japan) was used for statistical analysis.

## 3. Results

### 3.1. Patients

**Figure 1:**
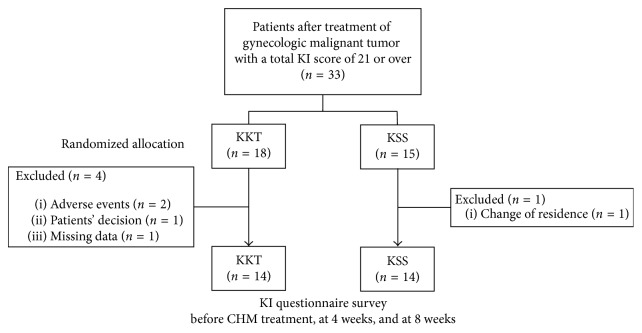
Patient flow. Patients who had general malaise after treatment of gynecological malignant tumor with a total score of 21 or over on the KI were randomly assigned to KKT or KSS. A Kupperman Menopausal Index (KI) questionnaire survey was conducted on subjective symptoms before tumor treatment, at baseline (before Kampo treatment), at 4 weeks, and at 8 weeks. Changes in KI score were evaluated in both groups. KI: Kupperman Menopausal Index; KKT: Kamikihito; KSS: Kamishoyosan; Kampo: Japanese Kampo medicine.

A total of 33 patients were enrolled: 18 in the KKT group and 15 in the KSS group (Figure 1). Three patients in the KKT group discontinued the study drug due to AEs (*n*=2) and the patient's decision (*n*=1), and one patient in the KSS group withdrew due to change of residence. Data at 4 weeks were missing for one patient in the KKT group. These five patients were excluded so the efficacy analysis was performed for 28 patients: 14 in the KKT group and 14 in the KSS group.  Table 2 shows the patient background. The mean age was 45.9 in the KKT group and 44.2 in the KSS group. Fifteen (83%) and 12 (80%) patients had undergone bilateral salpingo-oophorectomy (BSO) in the KKT and KSS groups, respectively. No significant differences were found in the patient background between the groups.

### 3.2. KI Scores

**Figure 2:**
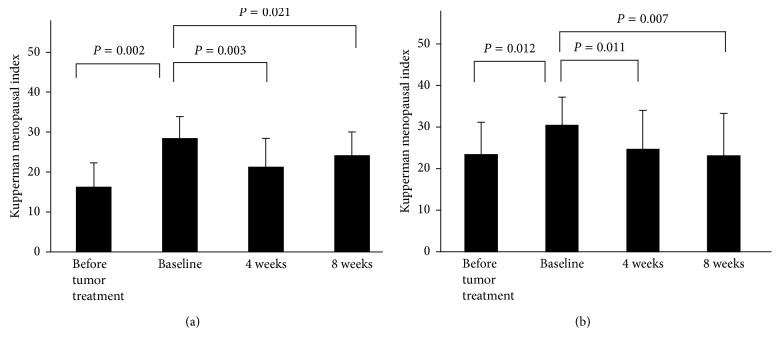
KI scores at four time points: (a) the KKT group and (b) the KSS group. The KI scores significantly elevated after initial tumor therapy (baseline) and significantly decreased at 4 and 8 weeks of study drug treatment compared with baseline in both groups. KKT: Kamikihito; KSS: Kamishoyosan.

Changes in KI scores are shown in Figure 2. The KI scores significantly increased after the initial anticancer therapy in both groups (KKT: from 16.3 ± 6.1 to 28.4 ± 5.5, *p*=0.0022; KSS: from 23.4 ± 7.8 to 30.4 ± 7.0, *p*=0.012). No significant difference was found in KI scores at pretreatment between the two groups. The KI scores significantly decreased at 4 and 8 weeks compared with baseline in both groups. In the KKT group, KI scores were 28.4 at baseline, 21.3 (*p*=0.009) at 4 weeks, and 24.1 (*p*=0.021) at 8 weeks. In the KSS group, those scores were 30.4, 24.6 (*p*=0.011), and 23.1 (*p*=0.021). No significant difference was found in ΔKI between the two groups (Table 3).  Table 4 shows the number of patients who complained of each subitem of KI and the changes in severity scores for both groups. In the KKT group, significant changes in severity score were found in not deepened sleep, dizziness and nausea, and heart palpitation. In the KSS group, significant changes were found in hot flash, sweating, not deepened sleep, dizziness and nausea, stiff shoulders/back and joint pain, and heart palpitation.

### 3.3. Safety

AEs were found in two patients in the KKT group: diarrhea (*n*=1; 5.6%) and joint pain (*n*=1; 5.6%). In both cases, the patient discontinued the study drugs. The causal relationships between the study drug and symptoms were not clear, although any drugs other than the study drug had not been prescribed.

## 4. Discussion

While the appearance of menopausal symptoms due to the loss of ovarian function caused by malignant tumor treatment is well known [17, 18], there have been no gold-standard therapies other than HRT. In the present study, we attempted to compare two different Kampo alternative therapies, KKT and KSS, and showed that the KI scores significantly decreased after Kampo treatment. While no significant difference in ΔKI between KKT and KSS groups was observed, each Kampo showed drug-specific efficacy in some of the subitems of KI. For example, KSS was more effective in patients with vasomotor symptoms and arthralgia/myalgia.

KKT is historically prescribed for treating symptoms of insomnia, restless sleep, nervousness, anxiety, giddiness, palpitations, easily fatigued, shortness of breath, and anorexia [19]. In the present study, KKT showed efficacy in the items of not deepened sleep, dizziness and nausea, and heart palpitation. These symptoms mostly correspond to indications for KKT. KSS is used to treat symptoms including depression, anger, irritation, headaches, flushed complexion associated with the autonomic nervous system, overexcitement, blushing, and chronic insomnia [19]. The present study showed efficacy in the items of hot flash, sweating, not deepened sleep, dizziness and nausea, stiff shoulders/back and joint pain, and heart palpitations in the KSS group. These results also mostly correspond to the indications for KSS. Efficacy against vasomotor symptoms such as hot flash and sweating was only found in the KSS group. These symptoms are thought to be associated with IL-6, IL-8, and MCP-1. As KSS has been reported to inhibit these cytokines and chemokine [20], this may explain the pharmacological effects of KSS. In the KKT group, efficacy was not found against fatigability, one of the indications for KKT. However, the reason might be due to the invasive surgery undergone in the KKT group. Therefore, based on the above findings, while both KKT and KSS are effective for insomnia, vertigo, and palpitation, KSS might be indicated preferably to the patients who have vasomotor symptoms such as hot flash and sweating, or arthralgia and myalgia. Given that the improvement in the symptoms almost corresponded to the indications of each study drug, KKT and KSS might be effective therapies for menopausal symptoms in patients with loss of ovarian function arising from gynecological tumor treatment, further indicating that tailor-made Kampo therapy might contribute to the improvement in patients' life after cancer treatment.

The present study has several limitations. First, this is a single-center trial. The sample size was small and insufficient to clearly distinguish the efficacy of each drug. Although we conducted a sample size calculation which resulted that 30 cases in each group would be needed for the comparison of the effect of KKT and KSS, it was not possible to recruit a total of 60 patients for several reasons during the study period: (1) a certain number of patients chose HRT to relieve their menopausal symptoms and (2) cancer patients seldom complained of menopausal symptoms since they had been always concerned of the recurrences. Second, it was not possible to use a placebo drug. This study was performed under health care services provided in Japan, and IRB of the institute did not allow us to use a placebo drug. Future studies with larger samples including placebo would help to identify the true effect of each Japanese Kampo medicine. Third, the purpose of this study was to analyze what type of Japanese Kampo medicine would be suitable to relieve menopausal symptoms after treatment of gynecological malignancy. Thus, in this study protocol, only two different Japanese Kampo medicines were chosen as a study drug and the efficacies of HRT were not assessed during the study period. Since HRT is “gold standard” to relieve menopausal symptoms of patients, it would be necessary to compare the effects of Japanese Kampo medicine with HRT in the future study. Fourth, no blinding was carried out in this study. Each Japanese Kampo medicine is powder drug packaged separately, and therefore, it was not possible to prescribe blinded drugs for the safety reason. Finally, there is no unified method to assess climacteric symptoms. We used KI in this study because it has been widely used internationally. Although KI includes some problems such as score overlapping or score weighting, our results indicate that KKT and KSS are effective for physical and mental symptoms caused by the treatment of gynecologic malignancy. Further study is needed to confirm our findings using other assessment tools.

In conclusion, KKT and KSS were effective against menopausal symptoms in patients with loss of ovarian function arising from gynecological tumor treatment. Kampo therapy may contribute to the tailor-made medicine to patients suffering from physical and mental symptoms.

## Figures and Tables

**Table 1:** Kupperman Menopausal Index: Abe modification [16].

Symptoms	Subitem	Severity				Weighted factor	Score
		Severe	Moderate	Mild	None		
Vasomotor symptoms	(1) Hot flash	3	2	1	0	4	
	(2) Sweating	3	2	1	0		
	(3) Sensitivity to cold	3	2	1	0		
	(4) Dyspnea	3	2	1	0		
Paresthesia	(5) Numbness extremities	3	2	1	0	2	
	(6) Insensitive extremities	3	2	1	0		
Insomnia	(7) Difficulty falling asleep	3	2	1	0	2	
	(8) Not deepened sleep	3	2	1	0		
Nervousness	(9) Excitable	3	2	1	0	2	
	(10) Nervous	3	2	1	0		
Melancholia	(11) Depressive mood	3	2	1	0	1	
Vertigo	(12) Dizziness and nausea	3	2	1	0	1	
Weakness	(13) Fatigability	3	2	1	0	1	
Arthralgia and myalgia	(14) Stiff shoulder/back and joint pain	3	2	1	0	1	
Headaches	(15) Headaches	3	2	1	0	1	
Palpitation	(16) Heart palpitation	3	2	1	0	1	
Formication	(17) Formication	3	2	1	0	1	

*Note*. Sum of the product of the maximum severity score of each symptom and weighted factor.

**Table 2:** Patient background.

	KKT group (*n*=18)	KSS group (*n*=15)
Age, mean	45.9	44.2

BMI (kg/m^2^), mean	22.0	21.9

Primary disease, *n* (%) (duplication)		
Ovarian cancer	7 (38%)	2 (13%)
Uterine cancer	5 (28%)	7 (47%)
Cervical cancer	5 (28%)	6 (40%)
Ovarian borderline malignant tumor	1 (6%)	
Carcinoma in situ of uterine cervix	1 (6%)	

Initial treatment		
Surgery (BSO)	15 (83%)	12 (80%)
Surgery without BSO	1 (6%)	2 (13%)
CCRT	2 (11%)	1 (7%)

Complications (duplication)		
Hypertension	1 (6%)	1 (7%)
Diabetes		1 (7%)
Depression	1 (6%)	
Renal cell carcinoma	1 (6%)	
Deep vein thrombosis	1 (6%)	
Crohn's disease	1 (6%)	
Systemic lupus erythematosus	1 (6%)	
Pulmonary embolism	1 (6%)	

BSO: bilateral salpingo-oophorectomy; CCRT: concurrent chemoradiotherapy.

**Table 3:** Comparison of the two groups using the Kupperman Menopausal Index (KI).

	KKT group	KSS group	*P*
Baseline	28.4 ± 5.5	30.4 ± 6.8	0.42
8 weeks	24.1 ± 6.0	23.1 ± 10.2	0.98
ΔKI	4.3 ± 6.0	7.4 ± 8.3	0.32

KI: Kupperman Menopausal Index; ΔKI: difference in KI score between baseline and 8 weeks.

**Table 4:** Change in symptoms.

Symptoms	Subitems of Kupperman Index	KKT group				KSS group			
		*n*	Baseline	8 weeks	*P*	*n*	Baseline	8 weeks	*P*
Vasomotor symptoms	(1) Hot flash	13	2.2 ± 0.9	1.6 ± 1.1	0.16	12	2.3 ± 0.8	1.5 ± 1.0	0.046^∗^
	(2) Sweating	14	2.3 ± 0.6	2.1 ± 0.7	0.48	14	2.4 ± 0.7	1.6 ± 0.9	0.031^∗^
	(3) Sensitivity to cold	7	1.7 ± 0.8	1.3 ± 1.1	0.26	13	2.2 ± 0.8	1.8 ± 0.6	0.23
	(4) Dyspnea	6	1.8 ± 0.8	0.8 ± 1.0	0.084	11	1.7 ± 0.8	1.3 ± 0.9	0.16
Paresthesia	(5) Numbness extremities	7	1.3 ± 0.5	0.7 ± 0.8	0.16	9	1.7 ± 0.5	1.2 ± 1.0	0.23
	(6) Insensitive extremities	2	1.5 ± 0.7	0.5 ± 0.7	0.16	10	1.3 ± 0.5	0.9 ± 0.9	0.21
Insomnia	(7) Difficulty falling asleep	12	2.1 ± 0.8	1.6 ± 1.0	0.058	9	1.9 ± 0.8	1.4 ± 0.9	0.16
	(8) Not deepened sleep	13	2.2 ± 0.8	1.5 ± 1.0	0.033^∗^	11	2.3 ± 0.5	1.6 ± 0.9	0.020^∗^
Nervousness	(9) Excitable	10	1.3 ± 0.5	0.8 ± 0.8	0.096	8	1.4 ± 0.7	0.9 ± 1.0	0.10
	(10) Nervous	12	1.6 ± 0.7	1.4 ± 1.0	0.53	11	1.9 ± 0.8	1.8 ± 0.9	0.74
Melancholia	(11) Depressive mood	11	1.7 ± 0.6	1.5 ± 0.9	0.37	11	1.9 ± 0.9	1.4 ± 0.9	0.13
Vertigo	(12) Dizziness and nausea	8	1.6 ± 0.7	0.5 ± 0.8	0.034^∗^	10	1.2 ± 0.4	0.3 ± 0.5	0.007^∗^
Weakness	(13) Fatigability	13	2.0 ± 0.7	2.1 ± 0.8	0.065	13	2.5 ± 0.5	2.0 ± 0.9	0.059
Arthralgia and myalgia	(14) Stiff shoulder/back and joint pain	11	1.8 ± 0.9	2.1 ± 0.9	0.32	14	2.5 ± 0.7	1.9 ± 1.2	0.038^∗^
Headaches	(15) Headache	10	1.7 ± 0.7	1.0 ± 1.2	0.17	8	1.9 ± 0.8	1.5 ± 1.2	0.41
Palpitation	(16) Heart palpitation	7	2.4 ± 0.8	1.6 ± 1.0	0.033^∗^	12	1.4 ± 0.7	0.8 ± 0.6	0.011^∗^
Formication	(17) Formication (itchy skin)	0	—	—	—	2	1.5 ± 0.7	1.0 ± 1.4	0.065

^∗^ *P* < 0.05.
